# Effects of cognac on coronary flow reserve and plasma antioxidant status in healthy young men

**DOI:** 10.1186/1476-7120-6-25

**Published:** 2008-06-03

**Authors:** Tuomas O Kiviniemi, Antti Saraste, Jyri O Toikka, Markku Saraste, Olli T Raitakari, Jussi P Pärkkä, Terho Lehtimäki, Jaakko J Hartiala, Jorma Viikari, Juha W Koskenvuo

**Affiliations:** 1 Department of Clinical Physiology and Nuclear Medicine, Turku University Hospital, Turku, Finland; 2 Department of Medicine, Turku University Hospital, Turku, Finland; 3 Department of Clinical Physiology, Tampere University Hospital, Tampere, Finland; 4 Laboratory of Atherosclerosis Genetics, Department of Clinical Chemistry, Tampere University Hospital, Tampere, Finland; 5 University of Tampere, Medical School, 33014 University of Tampere, Finland

## Abstract

**Background:**

The cardioprotective effects of certain alcoholic beverages are partly related to their polyphenol content, which may improve the vasodilatory reactivity of arteries. Effect of cognac on coronary circulation, however, remains unknown. The purpose of this randomized controlled cross-over study was to determine whether moderate doses of cognac improve coronary reactivity as assessed with cold pressor testing (CPT) and coronary flow reserve (CFR) measument.

**Methods:**

Study group consisted of 23 subjects. Coronary flow velocity and epicardial diameter was assessed using transthoracic echocardiography at rest, during CPT and adenosine infusion-derived CFR measurements before drinking, after a moderate (1.2 ± 0.1 dl) and an escalating high dose (total amount 2.4 ± 0.3 dl) of cognac. To explore the bioavailability of antioxidants, the antioxidant contents of cognac was measured and the absorption from the digestive tract was verified by plasma antioxidant capacity determination.

**Results:**

Serum alcohol levels increased to 1.2 ± 0.2‰ and plasma antioxidant capacity from 301 ± 43.9 μmol/l to 320 ± 25.0 μmol/l by 7.6 ± 11.8%, (p = 0.01) after high doses of cognac. There was no significant change in flow velocity during CPT after cognac ingestion compared to control day. CFR was 4.4 ± 0.8, 4.1 ± 0.9 (p = NS), and 4.5 ± 1.2 (p = NS) before drinking and after moderate and high doses on cognac day, and 4.5 ± 1.4, and 4.0 ± 1.2 (p = NS) on control day.

**Conclusion:**

Cognac increased plasma antioxidant capacity, but it had no effect on coronary circulation in healthy young men.

**Trial Registration:**

NCT00330213

## Introduction

Moderate consumption of alcohol is associated with reduced coronary artery disease mortality [1,2]. The cardioprotective effects of alcoholic beverages are partly related to their polyphenol content, which may be associated with improved vasodilatory reactivity of arteries [3,4]. A moderate (ethanol 0.5 g/kg) [5] and a high dose of red wine (ethanol 1.0 g/kg) has been shown to increase coronary flow reserve (CFR), but pure vodka and white wine had not such effect [3]. The effects of cognac on coronary circulation, however, have not been evaluated, although many elderly patients with coronary artery disease drink habitually cognac to releave cardiac symptoms.

The beneficial endothelial effects of polyphenol containing beverages, such as red wine, have been attributed to both ethanol and antioxidative polyphenols [6,7]. However, their relative contributions in vivo remain to be established. Cognac is known to contain polyphenols [8], but the bioavailability of the antioxidant content of cognac has not been previously addressed.

Cold pressor test (CPT) and CFR measurements are widely used methods to study coronary circulation [9-11]. Impairment of coronary artery reactivity as shown by insufficient vasodilation, or vasoconstriction in CPT, or decreased CFR occurs in various cardiovascular diseases, such as hypertension [12], diabetes [13], hypercholesterolemia [14], coronary artery disease [15], and even in otherwise healthy subjects with increasing waist to hip ratio [16]. Abnormal response in these tests is related to impairment in coronary vascular smooth muscle or endothelial function or both [9].

The purpose of this randomized controlled cross-over study was to determine with transthoracic echocardiography whether cognac ingested in moderate or high doses improve coronary vasodilatory responses in healthy humans. We also studied the bioavailability of antioxidants by determining the plasma antioxidant capacity.

## Methods

### Subjects and study protocol

The study included 23 healthy, non-smoking Finnish men (mean age 23 ± 1.8 years, body mass index 24 ± 2.3 kg/m^2^, total cholesterol 4.2 ± 0.8 mmol/l, HDL cholesterol 1.6 ± 0.41 mmol/l, LDL cholesterol 2.3 ± 0.65 mmol/l, triglycerides 0.77 ± 0.21 mmol/l and plasma glucose 5.0 ± 0.46 mmol/l). None of the subjects were taking any medication. The study was carried out in accordance with the Declaration of Helsinki (2000) of the World Medical Association and approved by the Ethics Committee of the Southwest Finland Health Care District. All subjects gave their written informed consent. We calculated sample size of 16 subjects with a known SD of 1.0 (for CFR) and an assumed difference of 1.0 between the interventions (α = 0.05, β = 0.80). To deal with the loss of data during the analysis, we enrolled 23 subjects.

**Figure 1:**
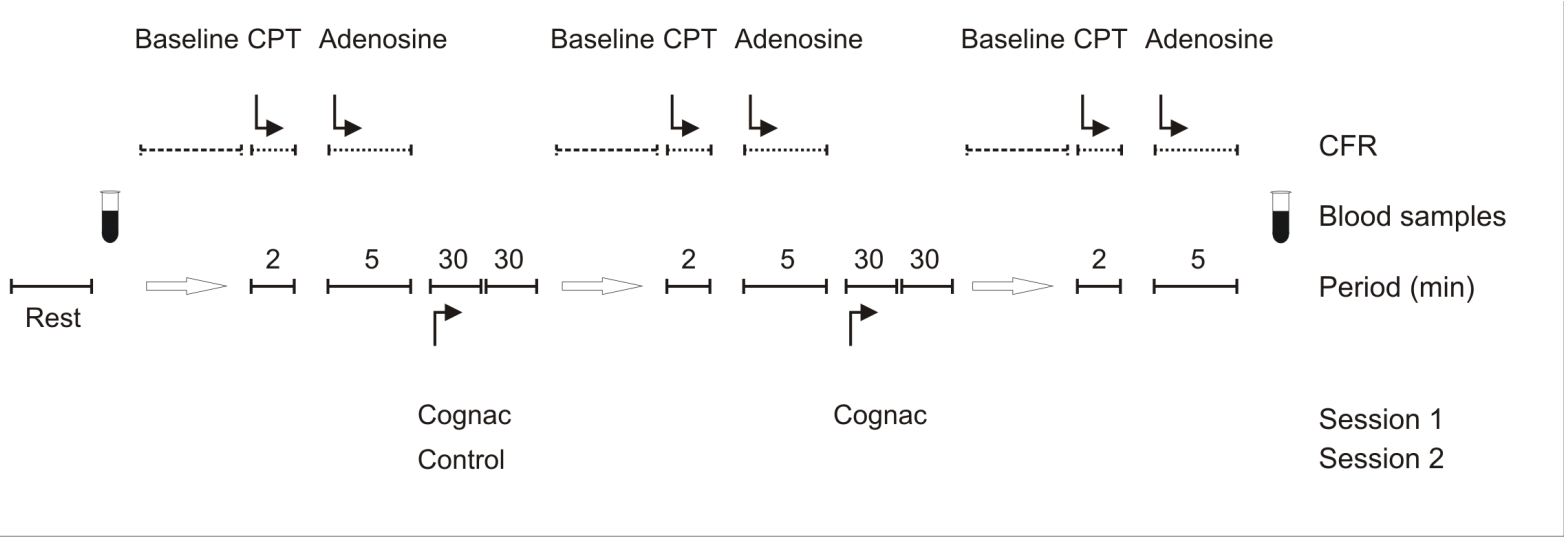
Study protocol. The subjects ingested two escalating doses of cognac (one dose 1.2 ± 0.1 dl). Each dose of cognac contained ethanol 0.5 mg/kg, and accordingly, the full dose of ethanol was 1.0 g/kg. CFR was measured 30 min after each dose. As a control, CFR was measured in randomized cross-over design without beverage ingestion twice.

Figure 1 outlines the study protocol. The subjects had an overnight fast. They were instructed to avoid caffeine for 12 h, and alcohol for 36 h before the studies. There were at least one week between the study days. The subjects were assigned to take two escalating doses of cognac. A moderate dose of cognac contained 0.5 g/kg of ethanol and a high dose 1.0 g/kg. Accordingly, the full doses of cognac were 2.4 ± 0.3 dl, respectively. CPT and CFR measurements were performed before drinking and again 30 minutes after each dose. As a control, the measurements were assessed in a random cross-over design repeatedly with 2 h interval when the subjects were not drinking. Blood pressure and ECG were monitored at rest and throughout the measurements. The rate pressure product was calculated as systolic blood pressure multiplied by heart rate.

### Cognac

The concentration of phenolic substances of the study cognac (Remy Martin VSOP, France) was determined according to the Folin-Ciocalteau method [17] (Sigma, St. Louis, MO). The total amount of phenolic substances given to the subjects was 100 mg ± 10 mg, including Ellagic acid 2.51 ± 0.28 mg, Hydroxybenzoic acid 0.70 ± 0.09 mg, Procyanidines (oligomers) 0.48 ± 0.06 mg, Hydroxycinnamic acids 0.12 ± 0.02 mg. In addition, the ability of the cognac to act as free radical scavengers against 1,1-diphenyl-2-picrylhydrazyl radical (Extrasynthèse, Genay, France) was tested spectrophotometrically [18]. The free radical scavenger capacitiy of the cognac was 7.0 ± 2.7%.

### Echocardiography

**Figure 2:**
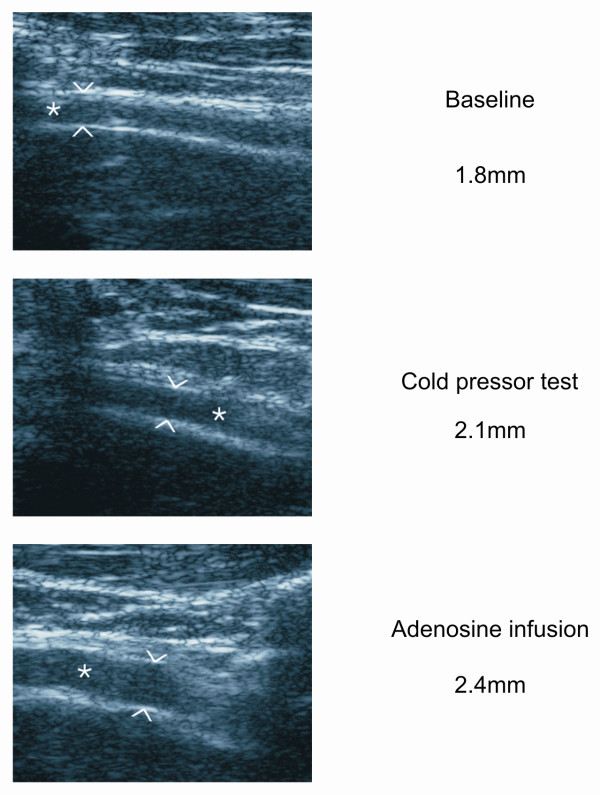
Epicardial coronary artery (LAD) diameter at baseline, during cold pressor test and adenosine infusion.

**Figure 3:**
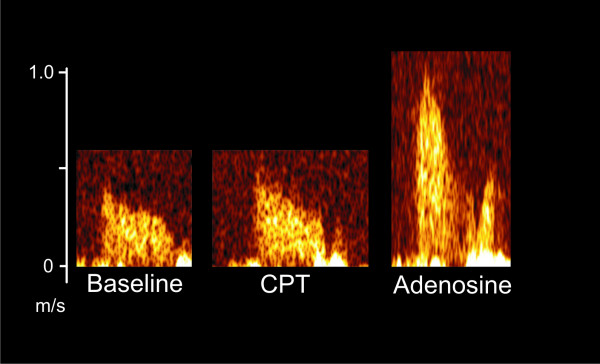
Coronary flow velocity in the LAD obtained using pulsed-wave Doppler at baseline, during cold pressor test (CPT) and adenosine infusion.

Coronary flow velocity and epicardial coronary artery diameter were measured in the distal left anterior descending coronary artery (LAD) with transthoracic echocardiography using the Sequoia C 512 ultrasound device (Acuson Inc., Mountain View, California, USA) with a linear 8.0 MHz transducer. The distal LAD was localized with color Doppler mapping using a modified apical two-chamber view scanning the interventricular sulcus as previously described [19]. The LAD flow velocity profile was recorded using a pulsed wave Doppler with angle correction. Moreover, the end-diastolic diameter of the LAD was measured in 2D images at the end of expiration from the largest distance between the luminal edges from the black-white interface of the near and far walls using ImageJ software (ImageJ 1.30 N, National institutes of Health, USA) [20]. The flow velocity profiles and 2D images of the LAD were obtained alternately at rest and throughout adenosine infusion as previously described (see Figures 2 and 3) [21]. Maximal mean diastolic velocities were used to calculate the percentage increase of coronary flow velocity during CPT to baseline and coronary flow velocity reserve (CFR) as previously described [19]. The variability of repeated CPT and CFR measurements was moderate (CV 13.4 ± 10.4 and 11.4 ± 8.9%, respectively), and the intra-observer variability was low (CV 3.0 ± 2.0 and 2.6 ± 4.0%, respectively), indicating small methodological and greater physiological variation as previously described [21].

All study data were stored in a digital workstation and analyzed offline in random order by an investigator blinded to the information of the day or the dose. In some studies, image quality was insufficient for analysis, and therefore, complete data could be recorded in 108/115 (94%) and 111/115 (97%) studies for CPT and CFR, respectively.

### Cold pressor test and CFR measurement

Baseline values were detected as an average of three separate diameter and three flow velocity measurements. After the baseline measurements, the subjects' right hand was placed into ice-cold water for 120 s. During the hand immersion, both diameter and flow velocity were measured in turn continuously. Epicardial coronary artery diameter during the CPT is expressed as an average of the measurements between 30 and 180 s and flow velocity as an average of the measurements between 10 and 150 s after placing the hand into the cold water. Minimum of three measurements was used. After hand immersion, there was at least 7 minutes interval before adenosine infusion. Intravenous adenosine (adenosin item 5 mg/ml, Item Development AB, Sweden) was infused at a rate of 0.14 mg/kg/min for 5 min and diameters and flow velocities were measured in turn continuously during the infusion. The results of the measurements are expressed as an average of three separate maximum diameter and flow velocity measurements.

### Blood samples

Venous blood samples were obtained for measurement of total cholesterol, LDL cholesterol, HDL cholesterol and triglyceride and glucose levels before the study. Serum ethanol concentrations were determined after the high dose of cognac using a standard photometric test (Modular P800, Roche Diagnostics GmbH, Mannheim, Germany). The antioxidative capacity of EDTA plasma was measured using a commercially available colorimetric assay kit (ImAnOx, Immundiagnostik, Bensheim, Germany) before drinking and after the high dose of cognac. The assay was done and results calculated according to the manufacturer's instructions. The lower detection limit of the assay was 130 μmol/l. The intra-assay coefficients of variation were 0.9% and 2.3% and the inter-assay coefficients were 1.63% and 2.43% for low and high levels, respectively. Optical density was determined using the Multiskan Ascent spectrophotometer at 450 nm (Thermo Labsystems, Helsinki, Finland). All samples were collected and stored in a similar manner and analyzed in randomized order.

### Statistical analysis

The data are presented as means ± SD. ANOVA for repeated measurements was used to assess differences of CPT and CFR between the doses. P-values indicating trend were calculated using the repeated measures regression analysis. A paired T-test was used to compare plasma antioxidant levels before and after beverage ingestion. The association of antioxidant capacity levels to CPT and CFR measurements was tested assessing the Pearson correlation coefficient between the differences in antioxidant capacity levels and the differences in CFR before and after drinking the beverages. P-values lower than 0.05 were considered significant. The statistical analyses were done using the Statistical Analysis System, SAS.

## Results

### CPT and CFR

**Figure 4:**
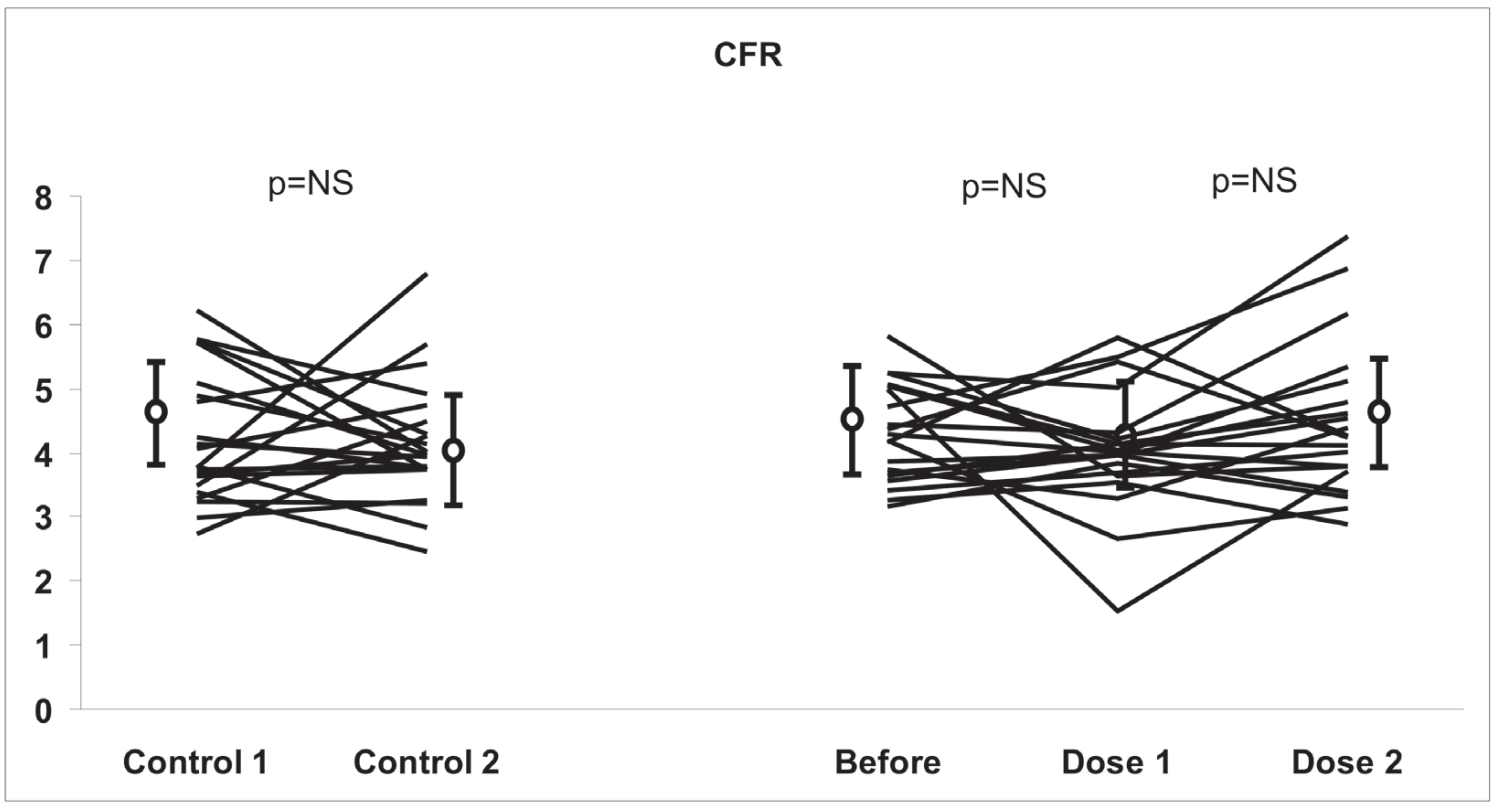
Individual changes in coronary flow velocity reserve (CFR) before cognac [before], after a moderate dose (ethanol 0.5 g/kg) [dose1] and a high dose (ethanol 1.0 g/kg) [dose2]. Control 1 corresponds to the first measurement on a control day. Control 2 corresponds to the measurement carried out 2 h after Control 1.

**Figure 5:**
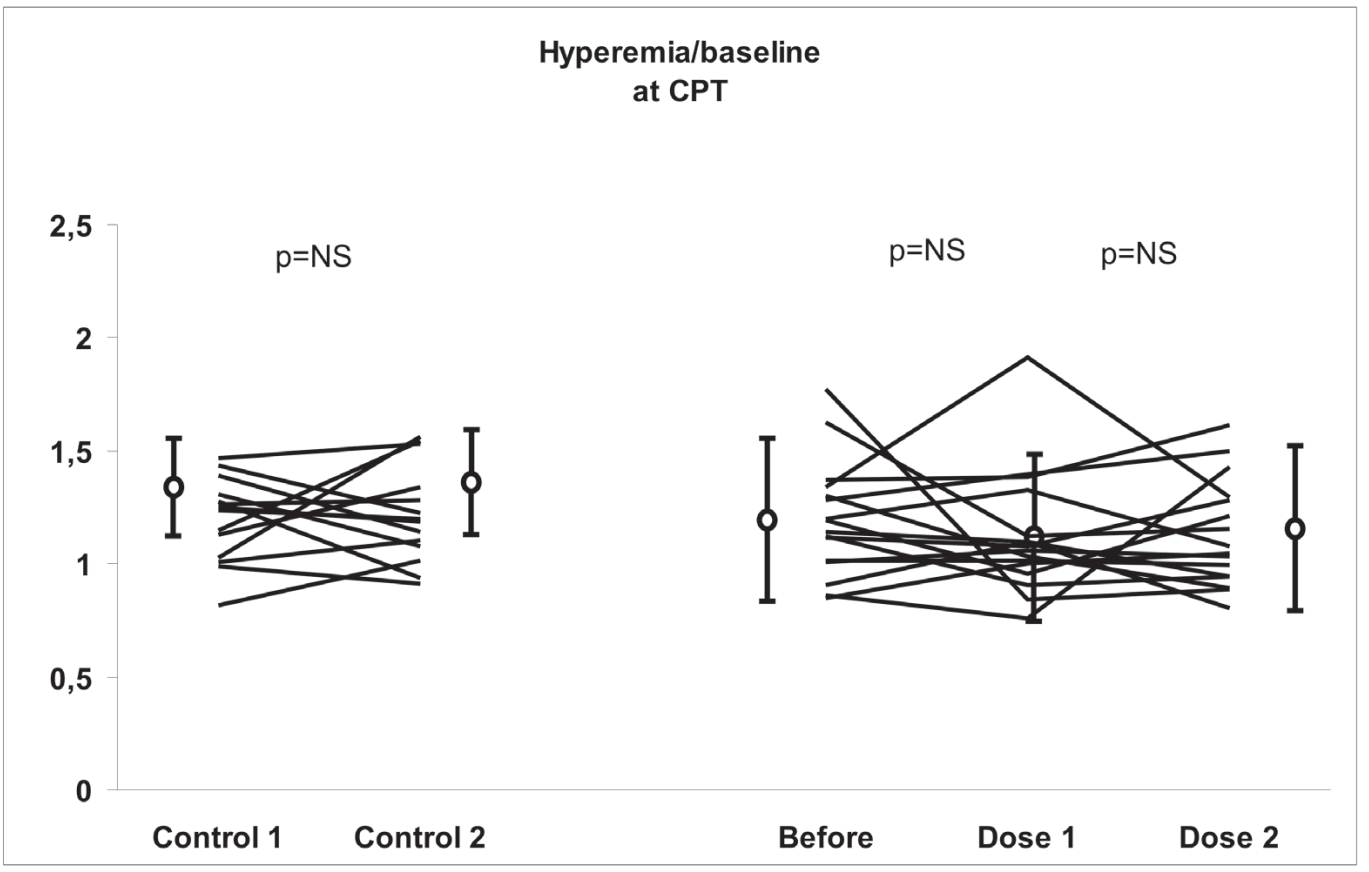
Individual changes in hyperemia to baseline ratio of flow velocity in cold pressor test (CPT). before cognac [before], after a moderate dose (ethanol 0.5 g/kg) [dose1] and a high dose (ethanol 1.0 g/kg) [dose2]. Control 1 corresponds to the first measurement on a control day. Control 2 corresponds to the measurement carried out 2 h after Control 1.

Table 1 shows the effects of cognac on coronary mean diastolic velocities and epicardial coronary artery diameter during CPT and CFR measurement. There were no significant difference between the before drinking and moderate and high doses values in coronary reactivity (see Table 2, Figures 4 and 5). The percentage increase in flow velocity during CPT was 21 ± 26%, 9 ± 30% and 17 ± 29% (p = NS) before drinking and after moderate and high doses, respectively, compared to control day (23 ± 20% and 25 ± 24%, p = NS). The corresponding values of CFR were 4.4 ± 0.8, 4.1 ± 0.9, and 4.5 ± 1.2 (p = NS) on cognac day, and 4.5 ± 1.4, and 4.0 ± 1.2 (p = NS) on control day. Moreover, the epicardial vasodilation was not significatly affected during CPT or CFR measurements before drinking and after moderate and high doses.

**Table 1:** Effects of cognac on mean diastolic velocity (MDV) and epicardial diameter of the LAD

		**Before drinking**			**Moderate dose**			**High dose**		
		Rest	CPT	Adenosine	Rest	CPT	Adenosine	Rest	CPT	Adenosine
**Cognac (n = 18)**	MDV (m/s)	0.20 ± 0.05	0.23 ± 0.07	0.85 ± 0.17	0.24 ± 0.07	0.25 ± 0.08	0.93 ± 0.20	0.22 ± 0.07	0.24 ± 0.08	0.91 ± 0.18
	Diameter (mm)	1.1 ± 0.4	1.2 ± 0.3	1.4 ± 0.4	1.1 ± 0.3	1.2 ± 0.3	1.4 ± 0.4	1.1 ± 0.3	1.1 ± 0.3	1.4 ± 0.4
										
**Control (n = 16)**	MDV (m/s)	0.21 ± 0.05	0.26 ± 0.06	0.93 ± 0.21	0.24 ± 0.06	0.29 ± 0.07	1.0 ± 0.25			
	Diameter (mm)	1.1 ± 0.3	1.2 ± 0.3	1.4 ± 0.3	1.1 ± 0.2	1.2 ± 0.2	1.5 ± 0.3			

n = number of subjects in a group. No significant changes were detected in corresponding values at rest, during CPT and adenosine infusion before drinking, and after moderate and high doses, respectively.

**Table 2:** Effects of cognac on coronary flow velocity during CPT (hyperemia to baseline ratio) and coronary flow velocity reserve (CFR) in the LAD

		**Before drinking**	**Before drinking vs. Moderate dose**	**Before drinking vs High dose**	**Trend**
**Cognac**	CPT	1.21 ± 0.26	-0.12 (-0.29 – 0.06) NS	-0.03 (-0.19 – 0.13) NS	P = 0.91
**N = 18**	CFR	4.4 ± 0.8	-0.29 (-0.83 – 0.24) NS	0.15 (-0.39 – 0.68) NS	P = 0.71

		Control 1	Control 2		
**Control**	CPT	1.23 ± 0.20	0.01 (-0.10 – 0.13) NS		P = 0.64
**N = 16**	CFR	4.5 ± 1.0	-0.45 (-0.99 – 0.09) NS		P = 0.85

Moderate and high dose correspond the amount of 0.5 g/kg and 1.0 g/kg ethanol, respectively. Data before drinking are presented mean ± SD. Data in the other columns are presented as the difference between the means (95% confidence intervals). The corresponding P value is from repeated analysis of variance. P-values indicating trend were calculated using the repeated measures regression analysis. Control 1 correspond the first measurement on control day. Control 2 correspond the measurement carried out 2 h after Control 1. N = number of subjects in a group. NS = not significant (P > 0.05)

**Figure 6:**
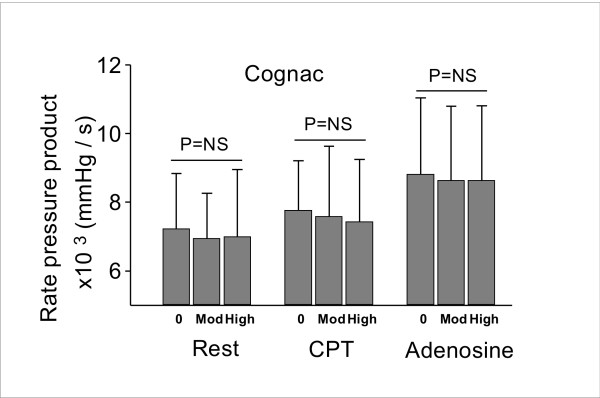
Effects of cognac on rate pressure product. No significant changes were detected in the corresponding values at rest, during CPT or CFR measurement before drinking, after moderate and high doses, respectively. 0 = before drinking; Mod = moderate dose; High = high dose.

Effects of cognac on rate pressure product are presented in Figure 6. No significant changes were detected in the corresponding values at rest, during CPT or CFR measurement before drinking, after moderate and high doses, respectively.

### Antioxidant capacity

Plasma antioxidant capacity increased from 301 ± 43.9 μmol/l to 320 ± 25.0 μmol/l by 7.6 ± 11.8%, (p = 0.01) after cognac. Serum alcohol levels increased to 1.2 ± 0.2‰ after the full doses of cognac. There were no significant association between the increase of plasma antioxidant capacity and coronary microcirculatory or epicardial vasodilation during CPT or CFR measurements.

## Discussion

We determined the effects of cognac on coronary reactivity, because epidemiological studies have indicated that any alcohol containing beverage may be beneficial for the heart [2]. In addition, many elderly patients with coronary artery disease use cognac to releave cardiac symptoms. It appeared that cognac had no effect on coronary reactivity in healthy young men, but it increased plasma antioxidant capacity. Furthermore, we found no association between the increase of plasma antioxidant capacity and coronary microcirculatory or epicardial vasodilation during CPT or CFR measurements.

Non-significant changes in CPT and CFR measurements reflect that cognac has no effect on coronary reactivity. Cognac contains antioxidative polyphenols, although in much smaller quantities than red wine [22], which is known to improve CFR [3,5]. In a recent *in vitro *study, the authors found that cognac induced vasodilation in the rat aorta [8]. Moreover, polyphenols from cognac are able to directly increase NO production without affecting O_2_^- ^and to enhance the capacity of bradykinin to produce NO in human endothelial cells [23]. We could not verify these findings in vivo in human coronary arteries. This can probably be explained by the low polyphenol content of cognac, which is insufficient to improve the reactivity to CPT or CFR. Moreover, cognac may contain too low levels of certain polyphenol subgroups, which are related to improvement vasodilatory reactivity. For example, oligomeric procyanidins of red wine have been shown to reduce vasoconstrictor endothelin-1 production in vitro [24]. Other polyphenol groups mediating improving vascular reactivity in vitro may include resveratrol, quercetin, and catechins [25]. It is also possible that the changes after cognac ingestion may be very small and, therefore, undetectable by the measurements. However, the variability of CPT or CFR measurements was moderate with low intraobserver variability and greater physiological variation. On the other hand, ethanol may blunt the vasodilatory effects cognac polyphenols [26].

We tested coronary vasodilatory responses by two distinct tests: CPT and CFR measurements. CPT is used as a physiological measure to assess coronary reactivity. In the CPT, the subject's hand or foot is placed in ice water, which produces a pain sensation. Nociception induces sympathetic stimulation and release of adrenalin and noradrenalin from the adrenal medulla [27-29], which increase heart rate, arterial blood pressure and myocardial oxygen demand. Increase in myocardial oxygen demand increases coronary blood flow. Moreover, sympathetic stimulation of α-adrenergic-receptors in the endothelium leads to the release of NO, which facilitates vasodilation of the resistance arteries [30]. Vasodilation of the resistance arteries increases blood flow in the conductance arteries as well, secondarily leading to vasodilation of the epicardial arteries by shear stress, despite α-adrenergic-mediated constriction of smooth muscle cells. In the normal heart, the net effect is vasodilation and increased coronary blood flow. In patients with endothelial dysfunction, a disturbance in vasodilator mechanisms leads to insufficient vasodilation, or vasoconstriction. CFR is defined as a ratio of coronary blood flow during maximal pharmacologically-induced vasodilation to baseline blood flow. Adenosine infusion is used to measure CFR, due to its capability to induce the maximal dilation of small coronary arteries. Secondarily, adenosine increases flow velocity in the epicardial arteries, also leading to flow-mediated vasodilation. Impairment of coronary artery reactivity as shown by decreased CFR occurs in various cardiovascular diseases, such as hypertension [12], diabetes [13], hypercholesterolemia [14] and coronary artery disease [15], and may be due to impairment in vascular smooth muscle or endothelial function or both.

Transthoracic echocardiography is a widely used non-invasive bedside method for assessing CPT and CFR responses and it correlates with invasive measurements using a Doppler guide wire [31] and myocardial blood flow quantified with PET [19]. This was the first time that changes in both coronary flow velocity and epicardial coronary artery diameter were measured simultaneously in a clinical trial using transthoracic echocardiography.

Some limitations of the present study should be pointed out. First, we studied young men only. However, as the purpose of the study was to determine the acute effects of cognac on coronary reactivity and plasma antioxidative capacity, using a homogenous sample may have diminished the role of confounding factors. Moreover, atherosclerotic processes begin early in life, and the attenuation of vasodilatory responses may be one of their early signs. Therefore, it makes sense to study subjects in this age group. Secondly, we saw marked variation in CFR (range 2.3 – 7.8) and moderate variability in totally reproduced CFR studies (CV 11.5 ± 8.7%), but intra-observer variability was low (2.6 ± 4.0%) indicating a small methodological error and greater physiological variation.

In conclusion, despite an improvement in plasma antioxidant capacity, cognac has no significant effect on coronary reactivity.

## Competing interests

The authors declare that they have no competing interests.

## Authors' contributions

TOK participated in the design of the study, acquisition, analysis and interpretation of data. He performed the statistical analysis and drafted the manuscript.

AS participated in the acquisition, analysis and interpretation of data. He participated in the drafting of the manuscript.

JOT participated in the acquisition and analysis of the data. He made a critical revision of the manuscript for important intellectual content.

MS participated in the acquisition of the data. He made a critical revision of the manuscript for important intellectual content.

TL performed the laboratory analyses of the plasma antioxidant capacity samples, participated in the interpretation of data and obtained funding. He made a critical revision of the manuscript for important intellectual content.

OTR participated in the design of the study, statistical analysis and interpretation of data. He made a critical revision of the manuscript for important intellectual content.

JPP participated in the acquisition of data. He made a critical revision of the manuscript for important intellectual content.

JJH participated in the design of the study and interpretation of data. He made a critical revision of the manuscript for important intellectual content, obtained funding and supervised the study.

JV participated in the interpretation of data. He made a critical revision of the manuscript for important intellectual content.

JWK participated in the design of the study, acquisition, analysis and interpretation of data. He participated in the drafting of the manuscript and supervised the study.

All authors read and approved the final manuscript.
